# A comparative analysis of health-related and individual quality of life in people with Parkinson’s disease

**DOI:** 10.3389/fneur.2025.1685649

**Published:** 2026-05-05

**Authors:** Ketevan Toloraia, Siegward Elsas, Arzu Tasci, Peter Fuhr, Ute Gschwandtner

**Affiliations:** Departments of Neurology and of Clinical Research, University Hospital Basel, Basel, Switzerland

**Keywords:** Parkinson’s disease, health-related quality of life, individual quality of life, anxiety, depression, apathy

## Abstract

Quality of life (Qol) results from a complex interplay of physical, mental, and social aspects. Traditional assessments of Qol may not adequately describe well-being in serious chronic illness such as in people with Parkinson’s disease (PwPD). This cross-sectional observational study explores differences and similarities of assessment through Health-Related Qol (HRQoL) versus individual Qol, measured as Schedule for the Evaluation of Individual Qol (SEIQoL), and analyzes the relative contributions of the most common psychological difficulties (anxiety, depression, apathy) in Parkinson’s disease. 48 PwPD (mean age: 67 years; IQR: 60.25–72.75; 12 women) completed SEIQoL and Parkinson’s Disease Questionnaires (PDQ-39) for HRQoL. In addition, measures for anxiety, depression, apathy, cognitive function, daytime sleepiness, and motor performance were obtained. Multiple linear regression was performed with both QoL summary indices as dependent variables. Interestingly, HRQoL and SEIQoL were not correlated to each other. Of all psychological difficulties only anxiety was a significant predictor of HRQoL and only self-rated apathy was a significant predictor of SEIQoL. High daytime sleepiness and medication doses were associated with lower HRQoL. In contrast, SEIQoL showed that family, relationships, autonomy and health were the most important domains, with health satisfaction being lowest. HRQoL and SEIQoL measure different aspects of QoL. Therefore, in future research studies both should be used together for an adequate assessment of therapies and effective rehabilitation strategies to ease the burden for PwPD.

## 1 Introduction

Parkinson’s disease is a neurodegenerative disease with one of the fastest growing prevalence (216 cases per 100,000 in 2050) and it projected to increase by 112%, reaching 25.2 million in 2050 (1). The manifestation of the disease, its progression and response to treatment is very individual (2). People with Parkinson’s Disease (PwPD) have a higher Disability-Adjusted Life Year (DALY) with respect to the general population (3). This means that the burden is significant due to the loss of healthy life years caused by the impact of the disease on both mortality and morbidity according to the Global Burden of Disease Study (1, 2). Parkinson’s disease is ranked third in the diseases with global disability adjusted life years per 100,000 people for all conditions with neurological health loss, behind stroke and Alzheimer’s disease (2). Despite these facts, there are still considerable knowledge gaps and stigma around disease perception and management (4). Existing research highlights significant gaps and stigma in disease perception and self-management in Parkinson’s disease, emphasizing the need for person-centered care and holistic assessment approaches. Demonstrate that psychological attitudes and self-perception play a major role in medication adherence (5).

In recent years, Qol has become a critical element in evaluating treatment outcomes and has been increasingly prioritized by healthcare systems across the world (6); and being utilized for informed policy decisions (7). Due to Parkinson’s Disease DALY ranking and its incurable and unpredictable nature (8), QoL is projected to remain an important consideration whilst setting goals of PD care and improving, promoting and maintaining well-being (3).

QoL is a multidimensional concept with different approaches to its assessment (3, 9, 10). It is a complex interplay of physical, mental, and social well-being, taking into account the cultural context, personal goals, expectations and concerns (11–13). It is important to note, in the context of this research paper, the term Schedule for the Evaluation of Individual Quality of Life (SEIQoL) (14) is also referred as individual quality of life (IQoL), and it is not referred to the Integrated QoL theory (15). In our opinion, assessing the QoL of PwPD, a distinction needs to be made between health related quality of life (HRQoL) and IQoL. The assessment of HRQoL in PwPD has long been the primary focus of clinical research and practice (16–18). The Parkinson’s Disease Questionnaire-39 (PDQ-39), developed by Peto et al. (19) is still a widely used instrument to assess HRQoL in PwPD by assessing eight specific domains: mobility, activities of daily living, emotional well-being, stigma, social support, cognition, communication, and bodily discomfort in managing their disease and the effectiveness of treatment throughout the course of Parkinson’s disease, from the first onset of symptoms to long-term follow-up. IQoL, on the other hand, takes a more personalized approach and has been utilized much less than HRQoL (14). Tools such as the Schedule for the Evaluation of Individual IQoL Direct Weighting (SEIQoL-DW) allow patients to measure their QoL from a subjective, individual perspective. During this assessment, respondents identify and prioritize five domains of life that they consider most important for their overall QoL and evaluate their current satisfaction in each area (20–22). The SEIQoL-DW is a recognized and validated instrument for individual QoL assessment and has been reviewed and “suggested” for use in Parkinson’s disease by the Movement Disorder Society task force. This tool highlights the importance of psychosocial domains and provides a more patient-centered understanding of QoL than conventional standardized measures (23, 24).

Person-centered care has become an increasing priority within the healthcare system, shifting the focus away from morbidity and mortality metrics toward broader measures of well-being (25). As clinical priorities evolved, the QoL of PwPD gained greater attention, emphasizing general well-being as a central aspect of care (26). As an indicator of a high QoL, well-being is focused on a needs-based approach, where QoL is determined by the extent to which a person’s individual needs and expectations are met (27). QoL reflects the difference between a person’s perception on their identity and disease management and their current experience at a given point in time (29).

Studies show that PwPD may perceive their condition differently depending on their resilience, the changes they perceive as positive related to the disease and other individual factors (28–30). Recent research also revealed how PwPD struggle on various factors such as navigating healthcare systems, inability to advocate for themselves, making decisions, and getting involved in their own care (31). Integrating HRQoL and IQoL measures can improve patient outcomes and ensure that care is patient-centered and holistic (3). This approach places the person rather than their disease at the center, promotes dignity and reduces stigma as both self-compassion and stigmatization are important factors influencing the psychological well-being of PwPD (32).

The present study aims to examine different elements of QoL in PwPD and to analyze the associations and differences between the PDQ-39 and the SEIQoL-DW. Furthermore, the study explores how these measures relate to depression, anxiety, apathy, cognition, motor performance, and daytime sleepiness in PwPD. This study addresses the following research questions:

1 How are HRQoL and IQoL associated with each other in PwPD?
2 How do mental health difficulties (depression, anxiety, apathy) and cognitive or motor performance relate to HRQoL and IQoL?
3 Do HRQoL and IQoL capture complementary or overlapping aspects of well-being in PwPD?

## 2 Materials and methods

### 2.1 Recruitment

62 PwPD were recruited from the Movement Disorders Clinic at the University Hospital Basel and via adverts. The participants took part in cognitive-behavioral group therapy (33) and high-frequency multimodal training (34). The present study utilizes data from these two intervention studies to pursue its own research objectives. Of the 62 recruited patients, ten were later excluded: three for personal and health reasons and four because they had participated in both studies. Four patients did not complete the SEIQoL-DW, resulting in a final sample of 48 patients for the analyses.

### 2.2 Data collection

Data were collected from March 2014 to July 2021. Inclusion criteria included a diagnosis of idiopathic Parkinson’s disease according to the UK Parkinson’s Disease Brain Bank criteria (35).

### 2.3 Inclusion and exclusion criteria

Exclusion criteria were moderate/severe dementia, insufficient German language skills, severe brain disorders and alcohol or drug dependence. All patients were on dopaminergic medication and tested in their “ON” state at participation. Exclusion criteria included insufficient knowledge of the German language and the presence of severe neurological diseases other than PD.

### 2.4 Ethics and informed consent

Under the approval of studies (verification number EKNZ: 294/13 and 2019/01834). All participants were fully informed about the nature of the studies and provided written informed consent.

### 2.5 Study design

This cross-sectional study, in which 48 PwPD participated, was designed to compare their HRQoL and IQoL and to identify possible influencing factors on both QoL measures. The primary outcomes were to analyze the PDQ-39 and SEIQoL and compare their summary index. Secondary outcome variables included global cognitive scores, psychological and neurological variables, and their influence on QoL. In addition, demographic information such as age, gender, education level, disease duration and medication dose were collected to obtain a comprehensive overview of the patient cohort and analyze these factors as potential confounders.

### 2.6 Assessment of QoL

#### 2.6.1 Health-related quality of life (HRQoL)

HRQoL was assessed using the PDQ-39. This comprehensive instrument contains 39 items from eight domains: Mobility, activities of daily living, emotional well-being, stigma, social support, cognitions, communication, and physical discomfort. Each item is rated on a five-point scale (0 = never, 1 = rarely, 2 = sometimes, 3 = often, 4 = always/not possible). The total score for each area ranges from 0 (no difficulty) to 100 (maximum difficulty), with lower scores indicating a better QoL. responses are converted into domain scores and a summary index. The summarized PDQ-39 index (PDQ-39SI) is the mean of the eight domain scores (19). PDQ-39 was developed and validated by Peto et al. (19).

#### 2.6.2 Individual quality of life (IQoL)

IQoL was assessed using the Schedule for the Evaluation of Individual Quality of Life Direct Weighting (SEIQoL-DW) instrument. This patient-generated measure requires individuals to identify and rate five life domains that they personally consider most important for their QoL. For each domain, respondents indicate their current level of satisfaction or functioning, then assign relative weights to reflect each domain’s importance. The SEIQoL-DW Summary Index (SEIQoLSI) is calculated by multiplying the satisfaction score for each domain by its assigned weight and summing these products. Total scores range from 0 (lowest possible QoL) to 100 (highest possible quality of life) (21, 22, 36). Acceptability, test–retest reliability, and validity of the SEIQoL have not yet been systematically evaluated in PwPD.

#### 2.6.3 Psychological and neurological assessment

##### 2.6.3.1 Motor symptoms

Motor symptoms were assessed using the Unified Parkinson’s Disease Rating Scale (UPDRS) Part III (37). Then the UPDRS Part III values were converted to MDS according to the method of Goetz et al. (38) which is considered as the current state of the art The MDS-UPDRS Part III (Motor Examination) consists of 33 scores derived from 18 items, each rated on a five-point Likert scale ranging from 0 (normal) to 4 (severe impairment). The total score ranges from 0 to 132, with higher scores indicating greater motor impairment. The MDS-UPDRS Part III is widely used in clinical trials and research studies to assess the severity of motor symptoms in PwPD (37).

##### 2.6.3.2 Anxiety symptoms

Anxiety symptoms were assessed using the German version of the Beck Anxiety Inventory (BAI) (39). The BAI comprises 21 questions, each of which is rated on a four-point Likert scale from 0 (not at all) to 3 (strongly). The total score ranges from 0 to 63, with higher scores reflecting a higher severity of anxiety. BAI has been validated by Leentjens et al. (40). A score of more than 13 indicates clinically significant anxiety in PwPD, as confirmed by Leentjens et al. (40).

##### 2.6.3.3 Depressive symptoms

Depressive symptoms were assessed using the German version of the Beck Depression Inventory-II (BDI-II) (41). This instrument comprises 21 questions, each of which is rated on a four-point Likert scale from 0 to 3. The total score ranges from 0 to 63, with higher scores reflecting more severe depression. The scores are categorised as follows: 0–13 (minimal), 14–19 (mild), 20–28 (moderate) and 29–63 (severe). A score above 14 indicates clinically significant depression in PwPD (42).

##### 2.6.3.4 Apathy symptoms

Apathy was measured using the German version of the Apathy Evaluation Scale (AES) (43). This scale consists of 18 items, each rated on a four-point Likert scale from 0 (not at all) to 3 (very strongly), resulting in a total score of 0 to 54. Scores ≥ 37 indicate clinically significant apathy in PwPD, a threshold that has been validated for this population. Self-rating (AES-S) and informant rating (AES-I) scales were used to better understand the patients’ condition. The self-assessment of apathy reflects the patient’s perception. In contrast, the informant-rated form provides an external view (44).

### 2.7 Cognitive function

Cognitive function was assessed using the Montreal Cognitive Assessment (MoCA), a commonly used instrument for screening mild cognitive impairment (MCI) in PwPD. It is a valid and reliable tool for screening MCI PwPD. MoCA scores range from 0 to 30, with scores below 21 indicating potential dementia (45).

### 2.8 Daytime sleepiness

Daytime sleepiness was assessed using the Epworth Sleepiness Scale (ESS) (46). The ESS is a self-administered questionnaire with eight items that are rated on a four-point scale (0–3). The total ESS score ranges from 0 to 24, with higher scores indicating greater daytime sleepiness. The ESS is a widely used instrument in clinical practice to assess general daytime sleepiness. It has been validated for use in various populations, including PwPD. We used a score of ≥ 10 to distinguish between the two groups: Patients with normal daytime sleepiness and patients with excessive daytime sleepiness (47).

### 2.9 Statistical analyses

Statistical analyses were conducted using IBM SPSS Statistics, version 28.0.1.0. Results are presented as medians and quartiles. Data distribution and normality were evaluated with the Shapiro–Wilk test. The BDI-II, PDQ-39 total score, BAI, and ESS scores were found to be non-normally distributed. Square root and Log10 transformations were applied to these variables to achieve normality. After transformation, the Shapiro–Wilk test yielded non-significant results (*p* > 0.05), indicating that the distributions of these variables did not deviate significantly from normality. Spearman rank correlation coefficients were used to analyse the correlations between PDQ-39SI, SEIQoLSI and other variables. The data were further analysed using a multiple linear regression model, including only those factors that were statistically significant in the correlation analyses. Multiple regression analyses with stepwise elimination were performed to identify significant predictors of QoL, using PDQ-39 and SEIQoL as dependent variables and adjusting for age and gender. Tolerance statistics and variance inflation factor (VIF) statistics were analyzed to assess multicollinearity between the independent variables (48).

In the exploratory data analysis, the SEIQoL aspects were assessed based on satisfaction and importance. A significance threshold of 5% (*p* < 0.05) with a 95% confidence interval (CI) was used in all analyses.

## 3 Results

### 3.1 Participant characteristics

Forty-eight PwPD (mean age 67 years, IQR 60.25–72.75; 12 women) participated in the study. The demographic and clinical data of the participants are shown in Table 1.

**Table 1:** Sample description.

Sample (*N* = 48)	Mdn [quantile]^a^
Age (years)	67 [60.25, 72.75]
Gender (female: male)	12:36
Education (years)	16 [13, 18]
Disease duration (years)	8 [4, 16]
LEDD, mg/day	474.85 [91.25, 687.5]
MoCA, points	25 [24, 27]
MDS-UPDRS III, points	20.7 [11.5, 27.02]
PDQ-39	19.61 [9.6, 29.34]
SEIQoL-DW	64.45 [55.4, 72.2]
BDI-II	7.5 [4, 10]
AES-I	28 [22.25, 34]
AES-S	30 [24, 36]
BAI	9 [3.25, 15]
ESS	6 [3, 10]

a All values represent median [quantiles]. Quantiles refer to 25th and to the 75th percentile.

LEDD, Levodopa-equivalent doses; MDS-UPDRS III, Movement Disorder Society – Unified Parkinson’s Disease Rating Scale, Motor Subscale; SEIQoL-DW, Schedule for the Evaluation of Individual Quality of Life – Direct Weighting; MoCA, Montreal Cognitive Assessment; BAI, Beck Anxiety Inventory; BDI- II, Beck Depression Inventory-II; AES-I, Apathy Evaluation Scale – Informant-rated; AES-S, Apathy Evaluation Scale – Self-rated; ESS, Epworth Sleepiness Scale.

### 3.2 Correlations between quality-of-life measures

There were significant correlations between the PDQ-39SI and the following variables: depression (*r* = 0.34; *p* = 0.02), anxiety (*r* = 0.66; *p* < 0.001), self-rated apathy (*r* = 0.37; *p* = 0.01), daytime sleepiness (*r* = 0.31; *p* = 0.02) and Levodopa equivalent daily dose (*r* = 0.27, *p* = 0.04). However, significant correlations were observed between the SEIQoL-SI and the anxiety (*r* = −0.37, *p* = 0.01), as well as between the SEIQoL-SI and the self-rated apathy (*r* = −0.41, *p* = 0.01) (Table 2). No statistically significant correlation was found between the SEIQoLSI and the PDQ-39SI scores (*p* = 0.08).

**Table 2:** Correlations (Spearman’s rank).

Tests	PDQ-39	SEIQoL-DW	BDI-II	AES-I	AES-S	BAI	ESS	MoCA	MDS-UPDRS-III	Dis. Duration	LEDD	Age	Education
PDQ-39	1												
SEIQoL-DW	−0.076	1											
BDI-II	0.345*	−0.27	1										
AES-I	0.027	−0.041	0.099	1									
AES-S	0.372*	−0.406**	0.24	0.501**	1								
BAI	0.655**	−0.370*	0.580**	−0.031	0.286*	1							
ESS	0.311*	−0.183	0.357*	0.008	0.351*	0.381**	1						
MoCA	0.145	0.078	0.1	−0.063	0.337*	0.041	0.054	1					
MDS-UPDRS-III	0.234	−0.296	0.432**	−0.124	0.164	0.374*	0.272	0.123	1				
Dis. Duration	0.084	−0.029	−0.07	−0.058	0.272	−0.14	0.166	0.261	0.171	1			
LEDD	0.296*	−0.193	0.299*	0.048	0.156	0.354*	0.1	0.162	0.295*	0.075	1		
Age	−0.117	0.038	−0.056	−0.231	−0.249	−0.074	0.223	−0.177	0.105	0.234	−0.003	1	
Education	−0.198	0.057	0.139	−0.09	0.027	−0.107	0.144	0.093	0.025	0.317*	−0.22	0.294*	1

*p < 0.01, *p* < 0.05.

LEDD, Levodopa-equivalent doses; MDS- UPDRS III, Movement Disorder Society – Unified Parkinson’s Disease Rating Scale, Motor Subscale; PDQ-39, Parkinson’s Disease Questionnaire; SEIQoL-DW, Schedule for the Evaluation of Individual Quality of Life – Direct Weighting; MoCA, Montreal Cognitive Assessment; BAI, Beck Anxiety Inventory; BDI- II, Beck Depression Inventory-II; AES-I, Apathy Evaluation Scale – Informant-rated; AES-S, Apathy Evaluation Scale – Self-rated; ESS, Epworth Sleepiness Scale.

### 3.3 Predictors of subjective quality of life

To explore predictors of subjective quality of life, a forward stepwise regression analysis was conducted with the SEIQoL-SI as the dependent variable. This analysis revealed that apathy severity was the only significant independent predictor, with greater apathy associated with poorer individual quality of life (*β* = −0.363, *p* = 0.013). The model explained approximately 11.2% of the variance in SEIQoL-SI scores (adjusted R^2^ = 0.112). Notably, anxiety and other variables did not contribute significantly and were excluded from the final model. Examination of collinearity statistics showed no indication of multicollinearity (Tolerance = 1.00, VIF = 1.00), and review of residuals demonstrated a satisfactory model fit and approximately normal distribution, supporting the validity of these findings.

### 3.4 Predictors of health-related quality of life

In a separate regression analysis for health-related quality of life, the PDQ-39SI was entered as the dependent variable. Anxiety severity emerged as the sole significant predictor, with higher anxiety scores being strongly associated with worse quality of life (*β* = 0.683, *p* < 0.001). This model accounted for 45% of the variance in PDQ-39SI scores (adjusted *R*^2^ = 0.453). None of the other variables included in the stepwise procedure (AES-S, BDI-II, ESS, LEDD) were retained as significant predictors.

### 3.5 Statistical model assumptions and robustness

As with the previous model, indices of multicollinearity were within acceptable ranges (Tolerance = 1.00, VIF = 1.00), and assessment of residuals supported the appropriateness and robustness of the regression model.

**Figure 1:**
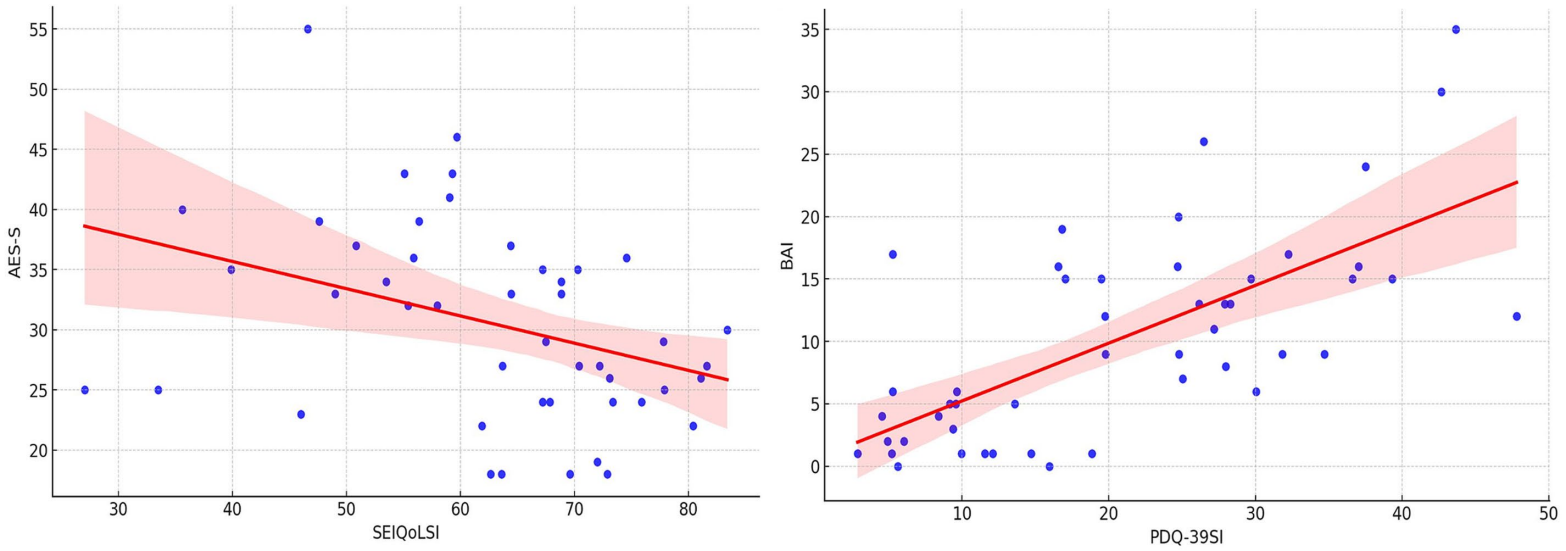
Correlations between individual quality of life and self-rated apathy as well as health-related quality of life and anxiety. SEIQoLSI - Schedule for the Evaluation of Individual Quality of Life, summary index; AES-S, Apathy Evaluation Scale – Self-rated; PDQ-39SI - Parkinson’s Disease Questionnaire, summary index; BAI, Beck Anxiety Inventory; *p < 0.05; **p < 0.001.

Together, these results indicate that within this cohort, apathy primarily drives reductions in subjective, individual quality of life, while anxiety exerts a substantial negative impact on health-related quality of life. Both regression models met key statistical assumptions, lending confidence to the interpretation of these associations (Figure 1).

### 3.6 Analysis of the PDQ-39 and SEIQoL domains and aspects

**Figure 2:**
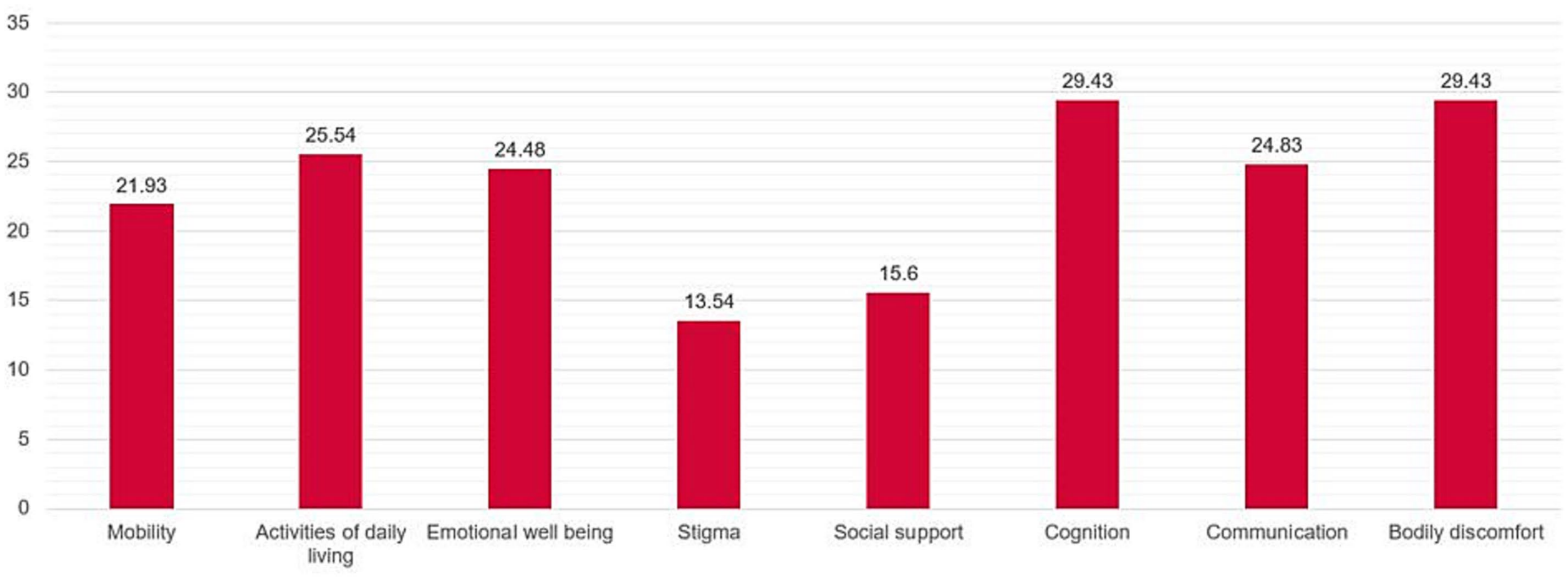
Domains of PDQ-39. The histograms show mean values of the PDQ-39 domains: (1) mobility, (2) activities of daily living, (3) emotional well-being, (4) stigma, (5) social support, (6) cognition, (7) communication, and (8) Bodily discomfort.

In the PDQ-39 domains, the areas of stigma and social support received the lowest scores, indicating better QoL in these areas. In contrast, the other six domains—mobility, activities of daily living, emotional well-being, cognition, communication, and bodily discomfort—had statistically higher scores, indicating poorer QoL in these areas (Figure 2).

When analyzing the possible correlations between PDQ-39 domains and the SEIQoL total score, significant negative correlations were found for the domains of mobility (*r* = −0.35, *p* = 0.01), activities of daily living (*r* = −0.35, *p* = 0.01), emotional well-being (*r* = −0.35, *p* = 0.02), and communication (*r* = −0.35, *p* = 0.01). Higher scores in these domains, indicating poorer QoL, were associated with lower SEIQoL-SI scores, which also reflect lower QoL.

**Figure 3:**
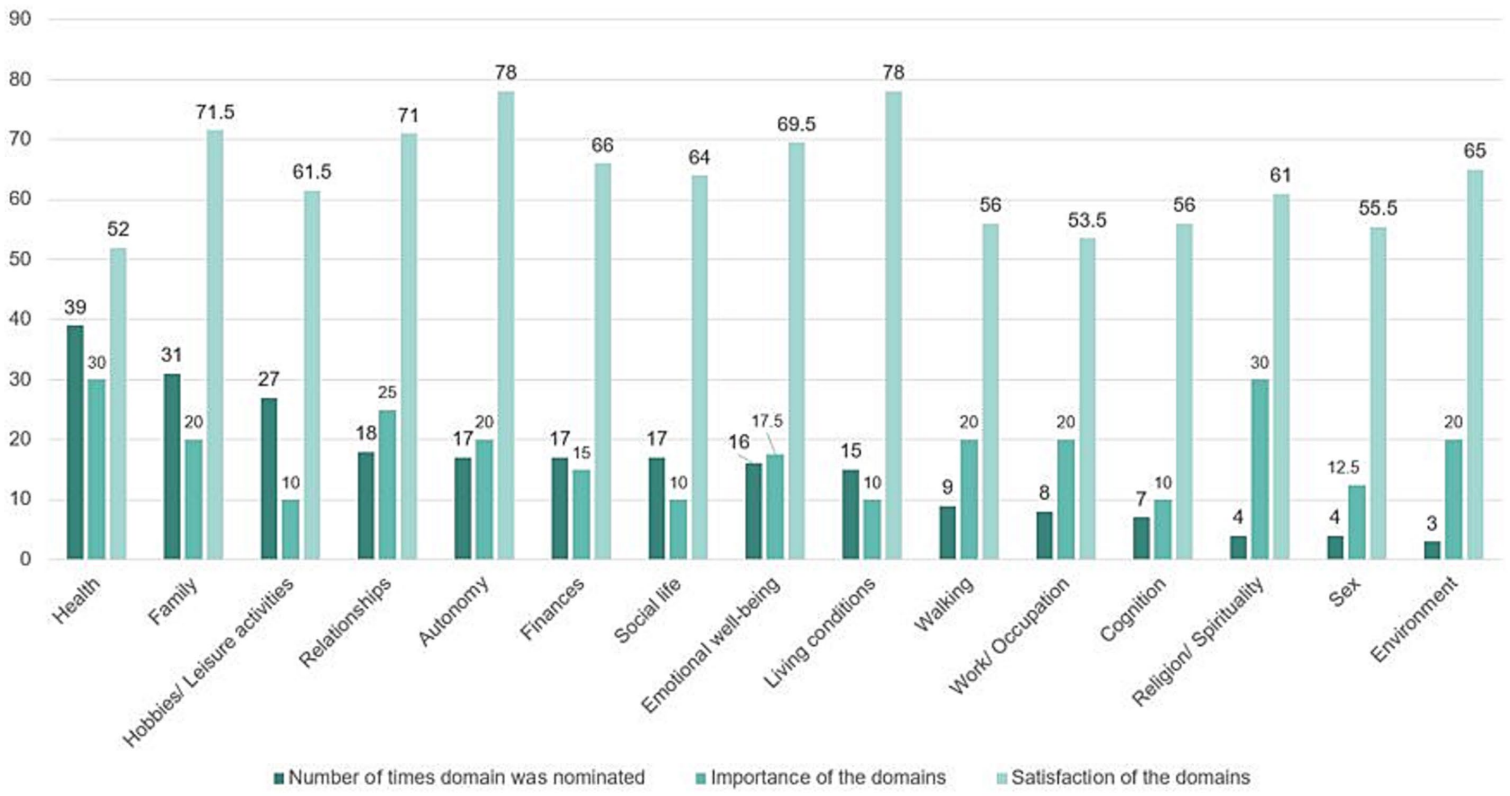
Individual quality of life factors identified by individuals using SEIQoL-DW. The histograms show the frequency (number of mentions), importance, and satisfaction of the SEIQoL-DW areas; Average values are provided for importance and satisfaction.

**Figure 4:**
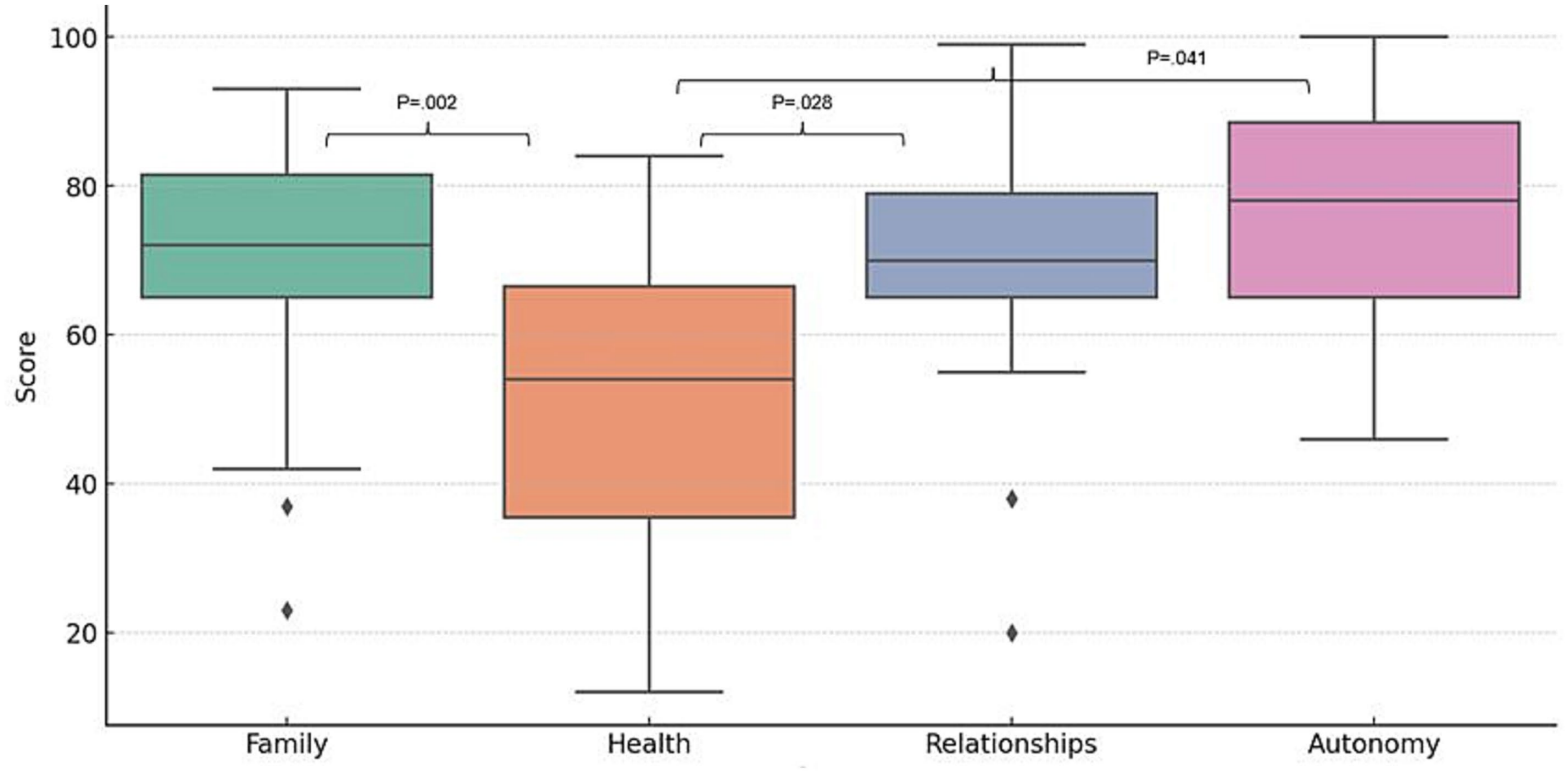
Boxplots of satisfaction with the most important aspects of individual quality of life.

Participants named ‘health’, ‘family’, ‘relationship’ and ‘autonomy’ as the most important areas of life. However, ‘health’ received the lowest satisfaction scores, while ‘family’, ‘relationship’ and ‘autonomy’ had significantly higher satisfaction scores (Figures 3, 4).

### 3.7 Correlations with PDQ-39 domains and demographic and clinical variables

A higher level of education was statistically significantly associated with lower communication skills. Perhaps individuals with higher education were more aware of their decrease in communication. Higher doses of Levodopa equivalent daily dose were associated with decreased mobility, emotional well-being, social support, communication skills and increased perceived stigmatization. Higher daytime sleepiness was associated with reduced mobility, activities of daily living, emotional well-being, and cognition.

## 4 Discussion

The aim of this study was to compare IQoL measured with the SEIQoL-WD with conventional HRQoL measured with the PDQ-39 and to investigate how they reflect psychological difficulties and neurological complaints.

### 4.1 Complementary insights from HRQoL and IQoL measures

In the present study, no statistically significant relationship was found between HRQoL and the IQoL indexes, suggesting that each measure provides a different insight into an individual’s well-being in this study cohort. Traditional QoL instruments tend to focus on the limitations associated with the disease (49).

The assessments were conducted on the “ON” phase of medication. The reasons were internal validity and feasibility. Standardizing the timing minimized interference from motor fluctuations and ensured that all participants could complete the assessments under comparable, stable conditions. However, this may have led to more optimistic assessments of quality of life (50) which may limit the ecological validity and transferability to everyday life.

### 4.2 Psychological predictors of quality of life

This study shows that anxiety is a significant predictor of HRQoL, while depression and apathy do not have the same influence. It is noteworthy that there was no significant prediction between SEIQoL and Anxiety. Anxiety is prevalent in PwPD and has a significant impact on HRQoL (51, 52). Anxiety in PwPD can lead to a significant worsening of physical symptoms, which in turn can increase anxiety (53). It is worth noting that the anxiety contains items that overlap with common motor symptoms of PD. Therefore, the motor symptoms of PD may significantly influence anxiety scores (54). This strong correlation supports the explanation that the PDQ-39 is a symptom-oriented questionnaire.

In the present study, self-rated apathy was significantly associated with both QoL scores. Previous studies have shown the influence of apathy on HRQoL (24, 55). However, we found that only the self-rated apathy was a significant predictor of the SEIQoL and not the PDQ-39. There was no association between the informant-rated apathy and the SEIQoL or the PDQ-39, indicating a difference between self-perceived and informant-rated symptoms. The perspective from which apathy is viewed can lead to significant discrepancies, even when the same questionnaire is used (56).

Spearman correlations revealed several significant correlations: the PDQ-39SI showed a significant positive correlation with the Levodopa-equivalent doses and the daytime sleepiness. Interestingly, these factors did not show a statistically significant impact on QoL when assessed with the SEIQoL. This discrepancy may be attributed to the holistic nature of SEIQoL assessments, in which other critical aspects, such as psychological and social factors, play a significant role (57).

Depression was significantly associated with the PDQ-39 but was not a significant predictor in our analysis. However, this may be due to our limited sample size. An earlier study by Lee et al. indicated that the PDQ-39SI is influenced by depression (58). Meta-analyses by Zhao et al. show mixed results regarding the influence of psychiatric comorbidities on the QoL of PwPD. Some studies indicate a significant impact of depression, anxiety, and sleep disorders, while others do not (59). It is important to note that some of these studies have only looked at HRQoL portion through PDQ-39 and do not have the same broad spectrum that our study presents. Our findings are consistent with the model of distress and protection, which states that QoL is determined by the balance between distressing factors (e.g., severe anxiety symptoms) and protective factors (e.g., social support) (60). These contradictions illustrate the complex interplay of factors that influence QoL in PwPD.

The lack of deterioration in QoL due to motor symptoms in our study can be explained by the greater influence of psychological difficulties in our cohort, which is consistent with findings in the literature (24, 61). In addition, people with chronic and life-threatening illnesses often readjust their internal frame of reference, a phenomenon known as response shift (62). Clinical research shows that patients adapt psychologically to their illness and shift their focus on QoL from physical deterioration to spiritual, psychological, and social domains. This adaptability emphasizes a person’s resilience in changing circumstances (63).

### 4.3 Analysis of quality-of-life domains

Health, family, relationships, and autonomy are key life domains in our cohort that were measured with the SEIQoL. However, the level of satisfaction with health is significantly lower than in other areas, which is consistent with the chronic health burden of Parkinson’s disease. From the individual’s perspective, QoL, health and functional status can be different constructs (64). Despite significant disabilities, PwPD can adapt to their new reality and report positive outcomes such as increased resilience and improved relationships (25, 65).

Within the PDQ-39 domains, stigma and social support received lower QoL scores, indicating better QoL. In contrast, the other six domains had statistically higher scores, indicating poorer QoL in these domains. Increased self-perceived social stigma in PwPD is associated with disease progression (66) and may be a determinant of QoL in PwPD (67). Schipper et al. emphasized the importance of social contact and living without social stigma due to PD. PwPD often face stereotypical perceptions of PD from others, further emphasizing the importance of social support and reducing stigma (68).

### 4.4 Study limitations

This study has some limitations: Our study has a small sample size. To maintain the sample size, we conducted a cross-sectional study instead of a longitudinal study, which means that we could only discuss associations and not causality. Another limitation may be that we used the BAI rather than the Parkinson Anxiety Scale (PAS), a scale that better captures anxiety in PwPD (69). In addition, in the present study, we did not use the UPDRS Part II and UPDRS Part IV, which could provide us with more information about motor experiences of daily living and motor complications as factors affecting the QoL of PwPD. Scores for anxiety and depression were relatively low in our study sample; however, we were interested to explore them as risk factors for a future worsening of motor impairment by Parkinson’s disease.

In the present study, we show that the HRQoL and the SEIQoL overlap and provide additional information that cannot be obtained with either approach alone.

### 4.5 Clinical implications

The clinical implications of quality of life in Parkison’s disease appear to be of enormous importance. The former COPPARDIS study (70) shows that the analysis of several quality of life measurements could be of great benefits in people with Parkinson’s disease. As shown in the literature (32) the stigmatization of patients with Parkinson’s disease is directly connected with the quality of life and should be supported in a more profound way (32). From a psychological level the literature has also shown that a feeling of control on the disease may have a large impact on the patient’s adherence (65).

### 4.6 Conclusion

In conclusion, health-related quality of life and individual quality of life represent distinct aspects of well-being in people with Parkinson’s disease and were not correlated in our study. These findings suggest that measuring both general daily functioning and personally valued life domains offers a more comprehensive understanding of patient well-being (58). Anxiety and self-reported apathy emerged as the most influential psychological factors, with anxiety being closely related to health-related quality of life, and self-perceived apathy to individual quality of life. In contrast, apathy as rated by informants did not correlate with either type of quality of life, highlighting the importance of capturing both self- and informant-based perspectives for a thorough assessment of motivational deficits. Daytime sleepiness and higher medication doses were significantly associated with health-related quality of life, but not with individual quality of life as perceived by patients. Furthermore, social support, autonomy, and freedom from stigma were identified as key contributors to well-being in this population. These results underline the need for future research and clinical interventions to assess both standard clinical and individualized quality of life, to address the full spectrum of challenges faced by people with Parkinson’s disease.

## Data Availability

The raw data supporting the conclusions of this article will be made available by the authors, without undue reservation.
